# Using glycine to recover valuable metals from lithium-ion batteries

**DOI:** 10.1038/s41598-025-24300-4

**Published:** 2025-11-18

**Authors:** Ehsan Tahan, Hassan Koohestani

**Affiliations:** Faculty of Materials and Metallurgical Engineering, Semnan University, Semnan, Iran

**Keywords:** Lithium-ion battery, Glycine, Oxalic acid, Lithium carbonate, Nickel oxalate, Chemistry, Environmental sciences, Materials science

## Abstract

In recent years, with the increasing use of lithium-ion batteries (LIBs), recycling their valuable metals has become very important not only from an environmental but also from an economic perspective. In this study, an effective and environmentally-friendly approach was proposed for leaching cobalt and lithium from LIBs by using glycine. Accordingly, after the initial preparation of lithium-ion batteries, in the first stage, the dissolution of the metals manganese, cobalt, nickel, and lithium in a glycine solution (1 M glycine concentration, 60 min, and 60 °C) was carried out. After investigating several effective parameters, different metals were precipitated from the solution in several stages. Using oxalic acid at pH = 2, nickel oxalate (NiC_2_O_4_) was precipitated from a glycine solution. Then, by increasing the pH to the alkaline range (about 8) with soda and ammonia, manganese and cobalt hydroxides were precipitated. Finally, by adding sodium carbonate, lithium carbonate was obtained. At each stage, the composition of the precipitate and the solution contents were determined by ICP, XRD, and FESEM analyses.

## Introduction

The increasing demand for lithium-ion batteries (LIBs) due to their high specific energy, excellent performance, and long cycle life has led to their mass production, and the number of lithium-ion batteries consumed is expected to increase significantly. The disposal of these batteries can lead to significant resource waste and environmental pollution due to the release of toxic chemicals, including heavy metals, organic solvents, and fluorine-containing compounds^1–4^.

It is predicted that by 2030, LIBs waste will reach 24 billion units^3^. Since these wastes contain valuable metals such as nickel, cobalt, lithium, manganese, etc., their recycling is of great importance^5^.

Currently, pyrometallurgy, hydrometallurgy, and biometallurgy are the common techniques for treating spent batteries^6–9^. The most significant advantages of hydrometallurgy over pyrometallurgy include the ability to be implemented on various scales, the recovery of a wider range of metals, higher efficiency, reduced pollution, and, in certain cases, lower implementation costs and higher extraction percentages^2,10^. Various inorganic and organic acids carry out dissolution in acidic environments. The inorganic acids used for dissolving lithium-ion batteries are generally sulfuric acid, nitric acid, and hydrochloric acid^9,11^.

Despite its low cost and excellent extraction ability, the leaching process using mineral acids, unfortunately, results in high corrosiveness, the need for advanced equipment, a lack of selective dissolution, and the creation of secondary pollution (such as SO_3_, Cl_2_, and NO_x_ gases)^2,11,12^. For this reason, the use of organic acids with low acidity, strong compatibility with complex systems, and higher dissolution and efficiency has recently been considered. So far, organic acids such as acetic acid, ascorbic acid, tartaric acid, citric acid, DL-malic acid, succinic acid, oxalic acid, and glycine acid have been investigated for the recovery of valuable battery metals^13–16^.

Glycine (NH_2_CH_2_COOH) is the simplest amino acid that has acidic and basic functional groups and exhibits different molecular shapes at different pHs. This inexpensive industrial material has been proposed as a suitable extractant for the extraction of various metals from various wastes^11,17^.

Chen et al.^11^ were able to recover about 97% of cobalt and 91% of lithium from waste lithium boron using glycine as a leaching agent under optimal conditions. Rautela et al.^17^ examined the dissolution of lithium over time, considering factors like pulp density, glycine concentration, and temperature. They showed that glycine, as a green extractant, increased leaching efficiency and reduced the need for mineral acids. Nayaka et al.^14^ studied cobalt recovery from spent LIB cathode materials using glycine, achieving more than 95% cobalt extraction with 0.5 M glycine at 80 °C for 6 h.

Given that a comprehensive study on the recovery of various metals from spent LIBs by glycine has not been conducted, in this study, the recovery of valuable metals (cobalt, manganese, nickel, and lithium) by glycine dissolution has been developed. The process has several steps, starting with dissolution in glycine acid and then, in different stages, the metals are separated one by one.

## Materials and methods

For this study, a battery pack (HUAWEI HB4W13.7v) and the chemicals listed in Table 1 were used.

**Table 1:** Details of the chemicals used.

Chemical	Formula	Purity (%)	Supplier/Company
Sodium Chloride	NaCl	99	Scharlau
Glycine acid	NH_2_CH_2_COOH	99	Scharlau
Nitric acid	HNO_3_	65	Merck
Oxalic acid	C_2_H_2_O_4_	99	Merck
Sodium hydroxide	NaOH	98	Merck
Ammonia	NH_3_	25	Merck
Sodium carbonate	Na_2_CO_3_	99.5	Neutron

### Preparation operation

First, the batteries were discharged using a 5% NaCl solution for 24 h. Then, the anode, cathode, and plastic layer were separated. The battery components were completely separated, including 11% plastic, 29% anode, 33% cathode, 22% steel and metal coating, and 5% other components. To separate the battery composition and decompose the adhesive and volatile substances, the foils were dried in an oven for 120 min at 60 °C and separated from the resulting powder.

**Fig. 1:**
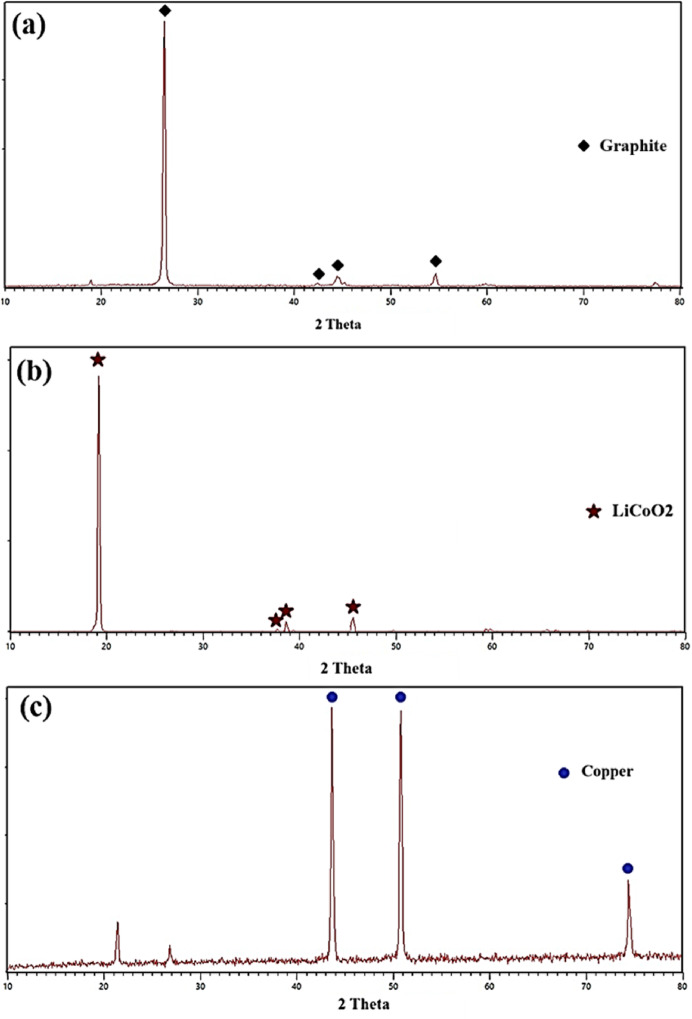
XRD patterns of different components of a lithium-ion battery: (**a**) graphite (JCPDS 26-1097), (**b**) cathode (JCPDS 50-0653), and (**c**) copper foil (JCPDS 003–1018).

The resulting powders were ground and mixed at room temperature for 5 min to homogenize, soften, and increase the efficiency of the leaching process. Then, using a vibrating sieve, a completely uniform sample with a particle size of 75 μm was prepared. The XRD pattern and the concentration of metals in the resulting powder are shown in Fig. 1 and Table 2, respectively.

**Table 2:** Concentration of metals in powder from lithium-ion batteries.

Metal	Conc. (%)
Li	5.8
Co	25.5
Mn	7.2
Ni	3.6

*Step 1:* Leaching.

**Fig. 2:**
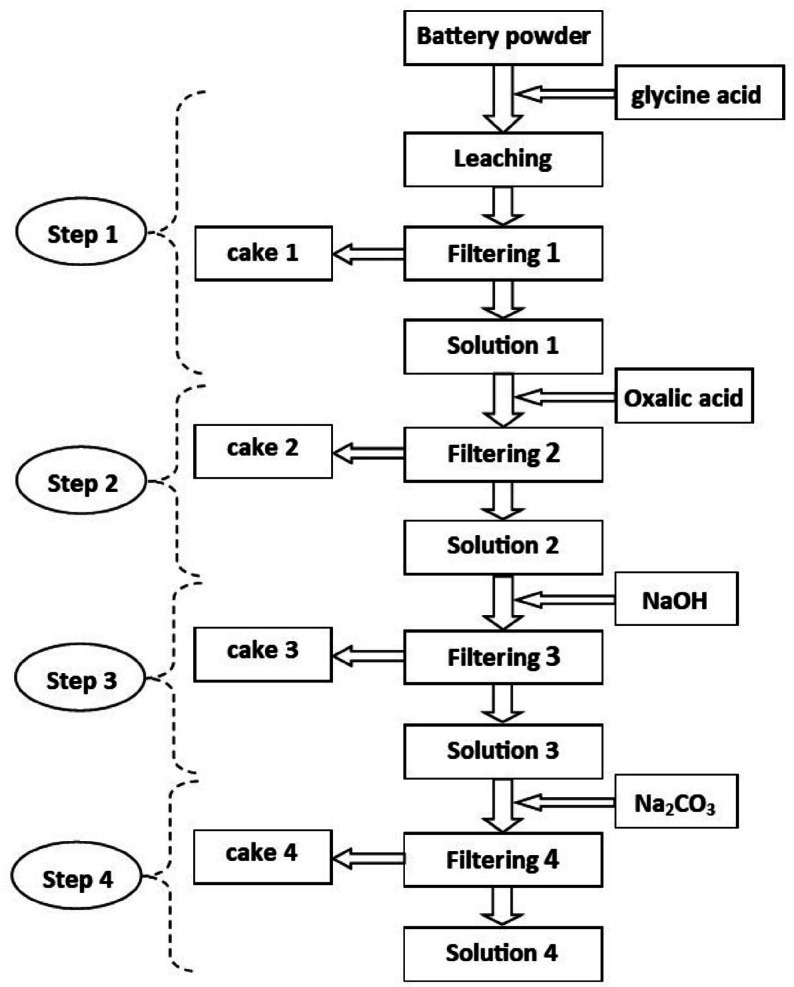
Flowchart of the different steps.

Leaching experiments were conducted in 100 mL solutions at varying concentrations using double-distilled water in an isothermal bath. At each stage of the leaching process and after filtration, the exact concentrations of lithium, cobalt, nickel, and manganese metals in both the solution and the solid were determined. Figure 2 shows the general procedure of the leaching experiment.

*Step 2*: Nickel separation

In the first stage, 25 g of battery powder was mixed and stirred in 100 cc of glycine solution with different concentrations at 60 °C for 60 min. The pH of the solution was 4, but to induce nickel precipitation, oxalic acid (C_2_H_2_O_4_) was added to the solution, lowering the pH to 2. Then, nickel precipitation was formed and settled after 6 h. The resulting solution was filtered, and the nickel precipitation was dried in a laboratory oven at 40 °C after being washed with distilled water.

*Step 3:* Cobalt and manganese precipitation

A certain amount of sodium hydroxide or ammonia (5M) was added to the remaining solution of the previous stage (in this case, the pH of the solution reached 8). With this action, the precipitation began to form and stopped after 6 h at 25 °C, and settled. The precipitation was removed from the solution by filtration and, after washing, dried in a laboratory oven at 70 °C.

*Step 4*: Lithium separation

Due to the high percentage of lithium dissolution in the glycine solution, 10 g of sodium carbonate (Na_2_CO_3_) was added to the remaining solution from the previous step at a temperature of 95 °C. At this time, the pH of the solution reached 9, and lithium carbonate precipitate began to form and settle after a few hours. The resulting solution was then filtered, and the undissolved material was separated from it. Finally, after washing in distilled water, it was dried in an oven at 70 °C to obliterate carbonate ions and impurities.

### Analysis methods

In this project, a Bruker Advance D8 X-ray diffraction device made in Germany, equipped with a nickel filter and a copper anode (CuKα˚) with a wavelength of 1.54056 Å, was used to identify the existing compounds. To determine the exact amount of metals in the solution, an ICP device with the Jenesis brand, made in Germany, was used. Finally, the obtained precipitates were analyzed by energy-dispersive X-ray spectroscopy (EDX). The precipitates of each stage were also evaluated using FESEM (field-emission scanning electron microscope, Tescan Mira3, Czech Republic), EDX, and map images.

## Results and discussion

**Fig. 3:**
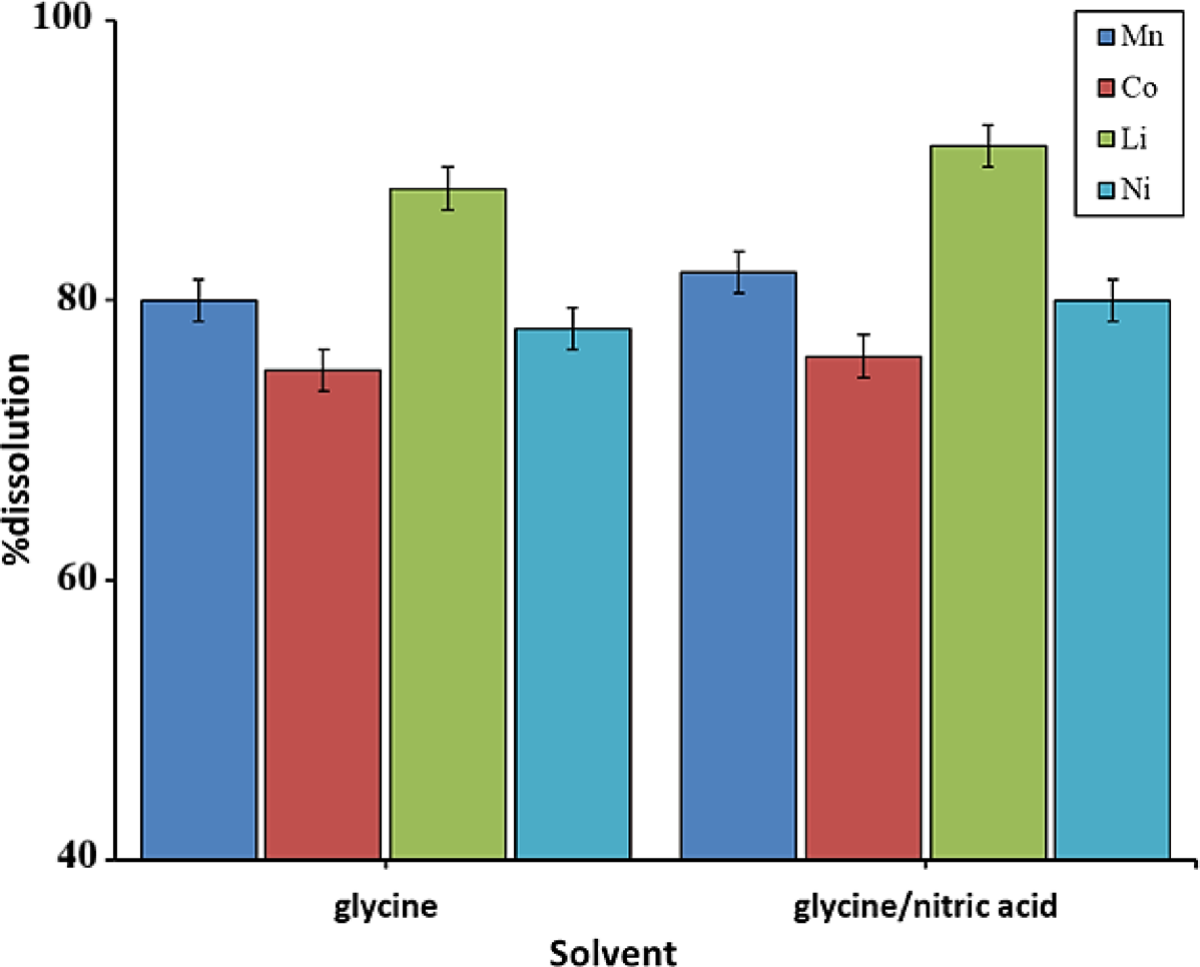
Effect of solvent type on the dissolution of various metals (1 M concentration, T = 60 °C, and t = 60 min).

Figure 3 shows the dissolution behavior of lithium-ion batteries in glycine acid and glycine/nitric acid mixtures under the same conditions. In glycine, lithium has the highest solubility (92%), while cobalt has the lowest solubility (73%); to some extent, the dissolution percentages of metals in these two acidic environments are similar. The primary objective of the leaching operation is to achieve high dissolution and selectivity. However, one of the most important factors in increasing dissolution is the reaction kinetics. The results show that the dissolution of some metals in the glycine/nitric acid mixture is somewhat higher than in the glycine medium. Although nitric acid has a higher dissolution power compared to glycine acid, it poses the same environmental hazards. Therefore, considering the acceptance of biocompatible methods in metal processing and recycling, glycine acid medium will be much more suitable. Battery dissolution in nitric acid will occur with the following reaction:

1 $${\text{LiCoO}}_{2} + {\text{HNO}}_{3} \Rightarrow {\text{LiNO}}_{3} + {\text{Co}}\left( {{\text{NO}}_{3} } \right)_{2} + {\text{H}}_{2} {\text{O}} + {\text{O}}_{2}$$

Nayaka et al.^18^ studied the recovery of cobalt from a lithium battery in a glycine/ascorbic acid solvent and showed that the addition of ascorbic acid did not cause a significant change in the cobalt dissolution efficiency. Orby et al.^19^ used the glycine leaching process to dissolve gold. According to studies, glycine can exist in aqueous solutions in three different forms: cationic glycine (N_3_NCH_2_COOH^+^) in acidic solutions, neutral zwitterion (H_3_NCH_2_COO^−^) in neutral solutions, and anionic glycinate (H_2_NCH_2_COO^−^). In addition to dissolving existing impurities, glycine can strongly adsorb metal ions such as gold, silver, nickel, manganese, etc. Eckstein et al.^20^ achieved 87% gold recovery using this method, using hydrogen peroxide instead of oxygen in glycine solutions to increase gold dissolution.

**Fig. 4:**
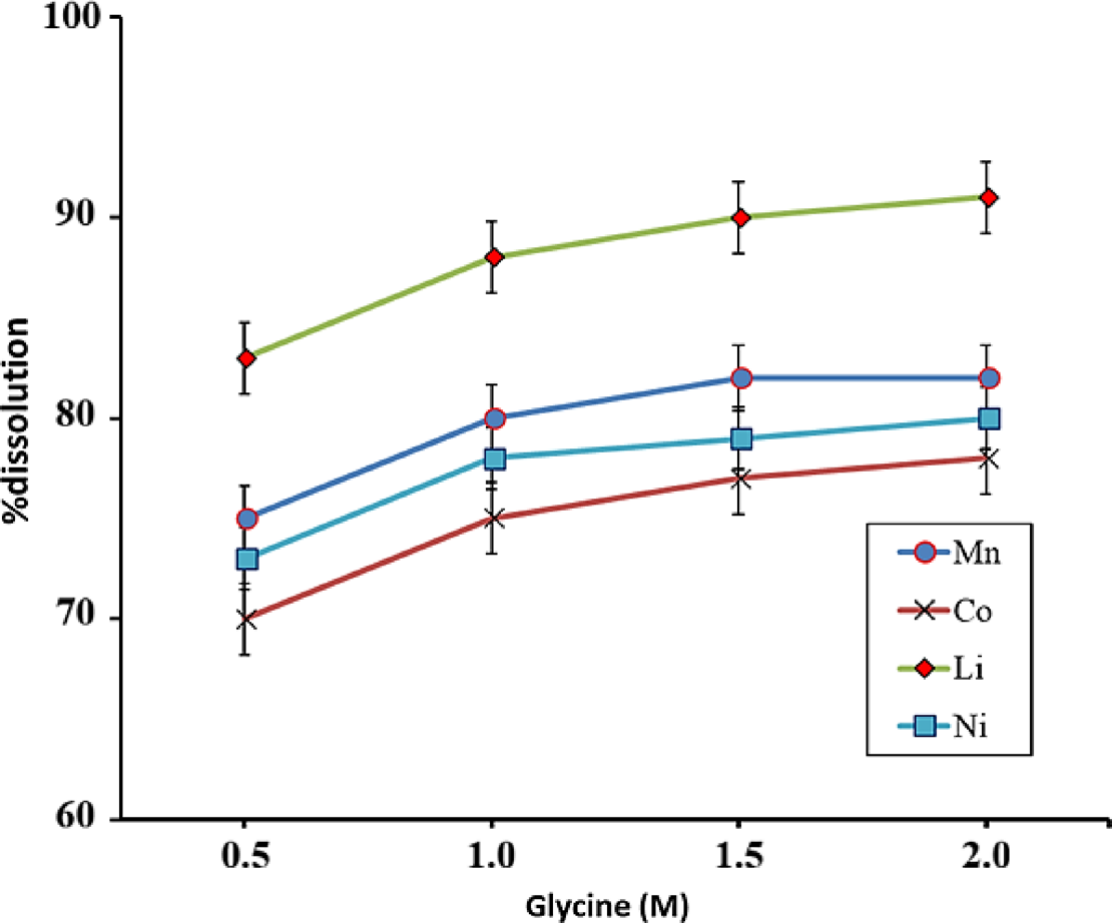
Dissolution behavior of battery powder at different concentrations of glycine (T = 60 °C and t = 60 min).

Figure 4 shows the effect of glycine acid concentration on the dissolution of metals at 60 °C and for 60 min. Acid concentration plays a significant role in metal leaching. Therefore, the leaching process was investigated at concentrations of 0.5 to 2 M with other parameters remaining constant. It is observed that by increasing the concentration of glycine acid from 0.5 to 1 M, the recovery of Mn, Co, Li, and Ni increases from 75%, 70%, 83%, and 73% to 80%, 75%, 88%, and 78%, respectively. It is obvious that under these conditions, the reaction kinetics will also increase. At concentrations greater than 1 M, the recovery of metals remains almost constant.

In similar studies, it has been demonstrated that under various dissolution conditions, the dissolution efficiency of metals remains nearly constant with an increase in glycine concentration above a certain threshold^18,21^.

Khadari et al.^22^ investigated the effect of glycine concentration on the dissolution of chalcopyrite, demonstrating that increasing the glycine concentration enhanced dissolution. However, this increase was noticeable at dissolution times above 6 h. Therefore, as can be seen from the reactions below, the formation of metal-glycine complexes is determined by many factors. High concentrations of glycine can enhance the ionization reaction of glycine, thus providing sufficient glycine ligands to increase the leaching of metal ions^11^.

2 $$\mathrm{NH}_{2} \mathrm{CH}_{2} \mathrm{COOH} \Rightarrow \mathrm{NH}_{2} \mathrm{CH}_{2} \mathrm{COO}^{ - } + \mathrm{H}^{ + }$$

3 $$\mathrm{NH}_{2} \mathrm{CH}_{2} \mathrm{COOH} + \mathrm{LiCoO}_{2} \Rightarrow \mathrm{NH}_{2} \mathrm{CH}_{2} \mathrm{COOLi} + (\mathrm{NH}_{2} \mathrm{CH}_{2} \mathrm{COO})_{2} \mathrm{CO}$$

In this reaction, glycine (NH_2_CH_2_COOH) reacts with lithium cobalt oxide (LiCoO_2_) to produce lithium glycinate (NH_2_CH_2_COOLi) and dicobalt glycinate [(NH_2_CH_2_COO)_2_Co]. Glycine is an amino acid consisting of an amino group (NH^2-^) and a carboxyl group linked by a single carbon atom. Lithium cobalt oxide is a transition metal oxide containing Li and Co. When glycine reacts with lithium cobalt oxide, the carboxyl group (COOH^-^) of glycine acts as an acid and donates a proton (H^+^) to the lithium ion in LiCoO_2_. This results in the formation of lithium glycinate. At the same time, the cobalt ion in LiCoO_2_ reacts with the amino group (NH^2-^) of glycine to form a complex molecule called dicobalt glycinate [(NH_2_CH_2_COO)_2_Co]^11,23,24^.

Tanda et al.^25^ studied the mineral behavior of copper oxide in glycine solutions at different concentrations. According to their theory, glycine has special chemical and physical properties, and increasing the concentration of glycine increases the dissolution and is very useful for forming stable complexes with metals. As a result, copper extraction is very easy by this method. Mengjin Chen et al.^11^ studied the leaching process by glycine acid and investigated its kinetics. According to the kinetic study, the leaching process is controlled by surface chemical reactions and activation, which results in the recovery of 97.7% cobalt and 90.95% lithium under optimal conditions.

To extract the nickel, by adding a small amount of 1 M oxalic acid to the solution of the previous step, the pH = 4 was reduced to pH = 3 and then to pH = 2. As a result, nickel precipitated through the following reactions^26^:

4 $${\text{H}}_{2} {\text{C}}_{2} {\text{O}}_{4(aq)} \Rightarrow {\text{HC}}_{2} {\text{O}}_{4(aq)}^{ - } + {\text{H}}_{(aq)}^{ + }$$

5 $$\mathrm{HC}_{2} \mathrm{O}_{4(aq)}^{ - } + \mathrm{H}_{(aq)} \Rightarrow \mathrm{C}_{2} \mathrm{O}_{4(aq)}^{2 - } + 2\mathrm{H}_{4(aq)}^{ + }$$

Reaction between oxalic acid and nickel:

6 $$\mathrm{C}_{2} \mathrm{O}_{4(aq)}^{2 - } + 2\mathrm{H}_{(aq)}^{ + } + \mathrm{Ni}_{(aq)}^{2 + } + 2\mathrm{OH}_{(aq)}^{ - } \Rightarrow \mathrm{NiC}_{2} \mathrm{O}_{4(aq)} + 2\mathrm{H}_{2} \mathrm{O}_{(aq)}$$

The precipitation of nickel oxalate is due to its low solubility. The precipitation of nickel oxalate in solution is as follows:

7 $$\mathrm{NiC}_{2} \mathrm{O}_{4(aq)} \Rightarrow \mathrm{NiC}_{2} \mathrm{O}_{4(S)}$$

Allen^22^ reported the precipitation of nickel by oxalic acid in two stages. In the first stage, the following reaction, which involves the formation of a soluble complex, occurs:

8 $$2{\text{Ni}}_{(aq)}^{2 + } + 2{\text{H}}_{2} {\text{C}}_{2} {\text{O}}_{4(aq)} + \left[ {{\text{Ni}}\left( {{\text{C}}_{2} {\text{O}}_{4} } \right)_{2} } \right] \Rightarrow 3{\text{Ni}}_{(aq)}^{2 + } + 4{\text{H}}_{(aq)}^{ + }$$

The equilibrium constant of the reaction is about 10^18^, which indicates the formation of the desired complex ion for longer periods and a lower precipitation rate. In the second stage, nickel precipitates as oxalate:

9 $$[{\text{Ni}}({\text{C}}_{2} {\text{O}}_{4} )_{2} ] = {\text{Ni}}^{2 + }_{(aq)} \to 2{\text{NiC}}_{2} {\text{O}}_{4} \cdot {\text{2H}}_{2} {\text{O}}_{(s)}$$

Evidence has shown that this stage occurs heterogeneously on the chamber wall. On the other hand, in the presence of excess oxalic acid, the stability of the complex decreases^27^.

**Fig. 5:**
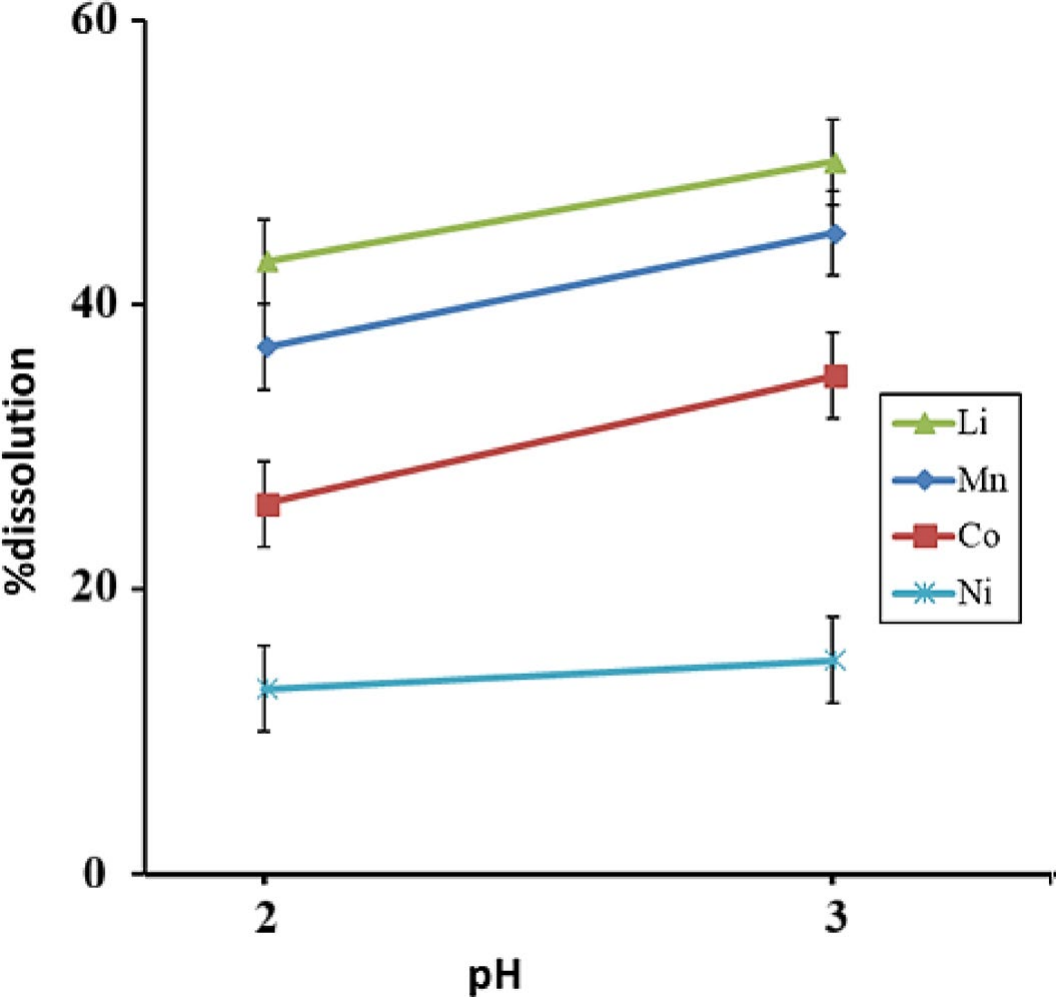
Dissolution behavior of battery powder in oxalic acid at different pHs (T = 60 °C and t = 60 min).

Figure 5 shows the dissolution of metals after the addition of oxalic acid at different pHs. At pH = 2, where the concentration of H^+^ in the solution increases, the concentration of nickel metal ion in the solution decreases. This is because dissolved nickel precipitates when sufficient C_2_O_4_^–2^ is present^28^. Meshram et al.^29^ showed by dissolving copper converter slag in oxalic acid that nickel has the lowest solubility in oxalic acid compared to cobalt, copper, and iron. Refly et al.^30^ also dissolved lithium battery cathode materials in ascorbic acid and then successfully precipitated nickel oxalate with oxalic acid.

**Fig. 6:**
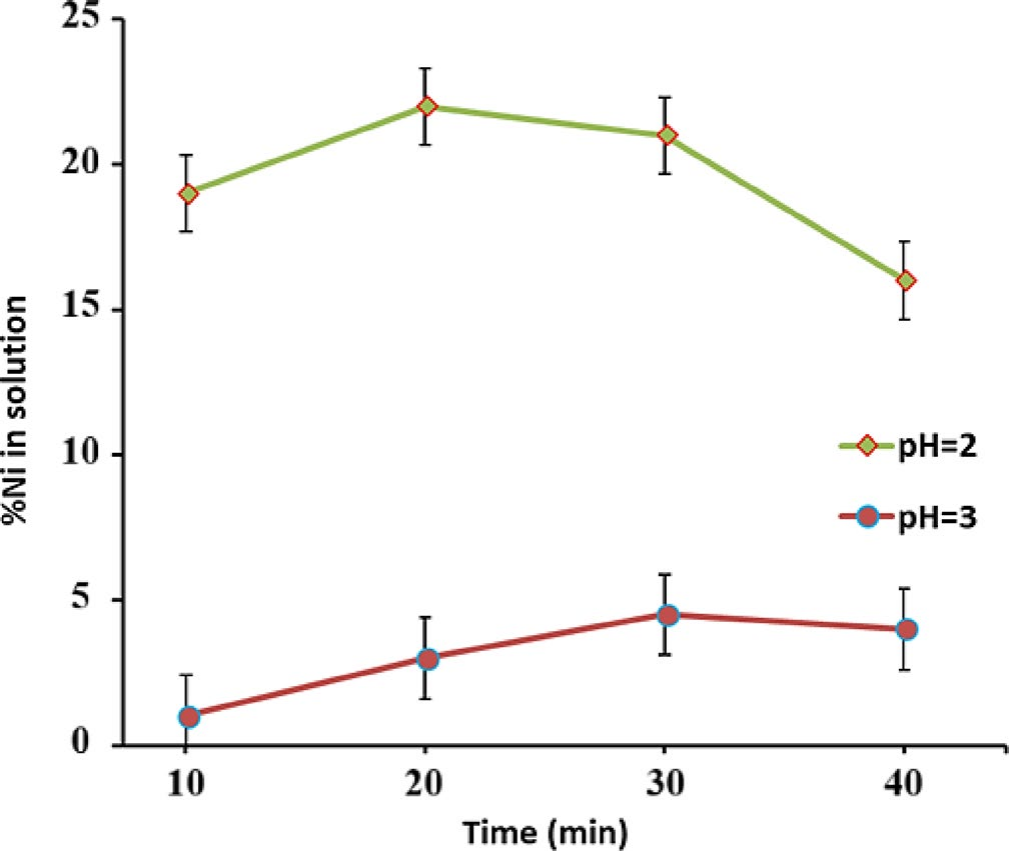
Nickel recovery over time at different pHs (T = 60 °C and t = 60 min).

Figure 6 shows the nickel recovery after adding oxalic acid at different pHs. Nickel recovery is strongly affected by pH. The recovery is increasing at 20–30 min, but at pH = 2, the recovery rate is high. The nickel concentration in the solution increased during the first 10 min of leaching, and then it decreased due to nickel oxalate precipitation. Increasing the time reduces the amount of nickel oxalate precipitation, which is why the recovery rate decreases after 30 min. The highest nickel recovery is obtained at pH = 2 and a washing time of 30 min, with a value of 22.25%.

**Fig. 7:**
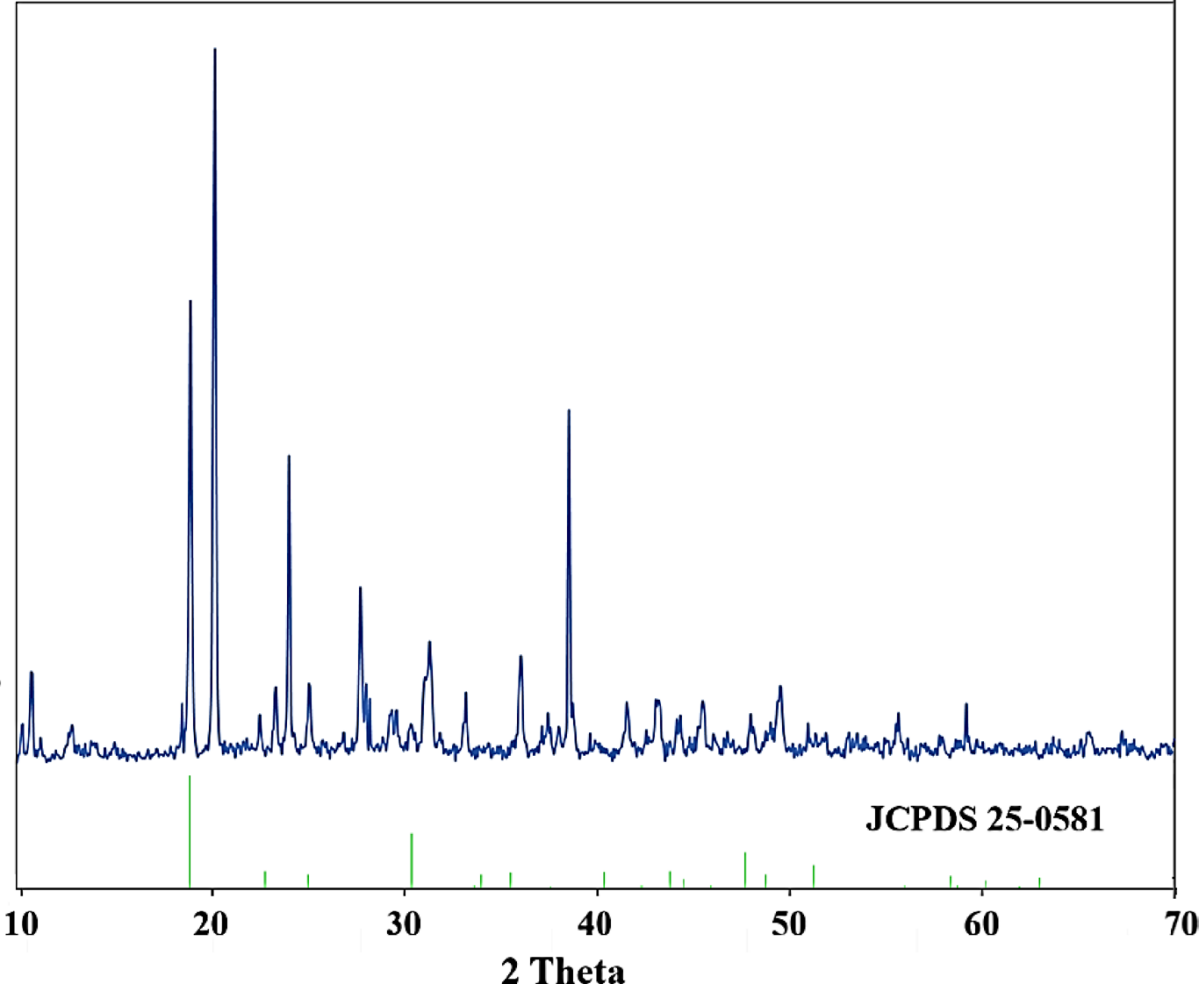
XRD pattern of nickel oxalate (NiC_2_O_4_) precipitate (JCPDS 25-0581).

**Fig. 8:**
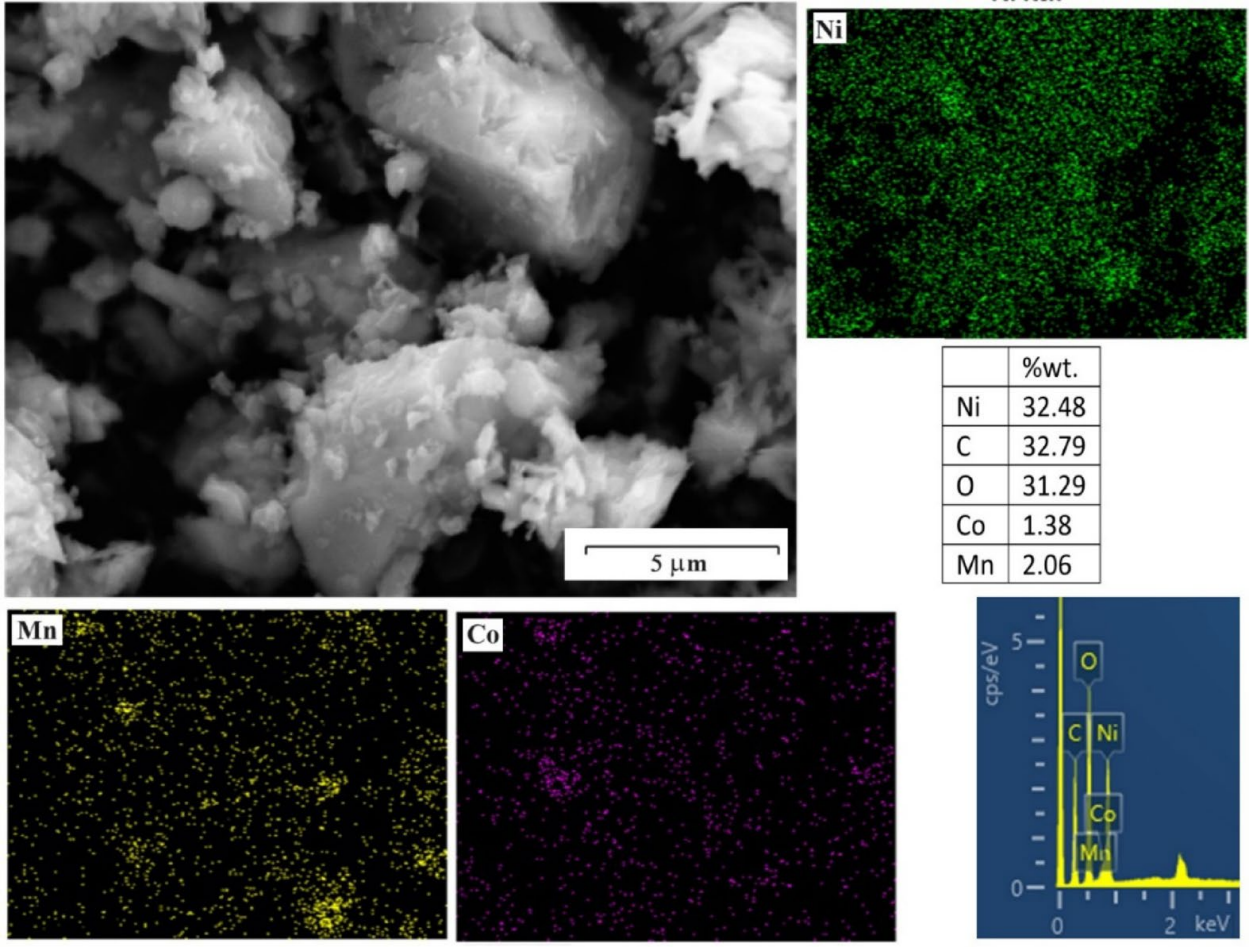
SEM image, MAP analysis, and EDX analysis of precipitated particles.

After removing the precipitate, the remaining solution was analyzed. Table 3 shows the metal concentrations in the solution for two cases: pH 2 and pH 3. It is evident that, despite the nearly identical levels of cobalt, lithium, and manganese in both cases, the nickel concentration has changed significantly. This indicates that pH influences the nickel precipitation process, with its recovery increasing as pH decreases. Although only a small amount of nickel remains in the solution, the other metals are still present. To confirm and identify the precipitate, the nickel precipitate was also analyzed after separation using XRD and FESEM techniques. Figure 7 displays the XRD pattern of the precipitate. According to JCPDS 25-0581, the peaks at angles 18.94, 20.18, 23.91, 27.56, 32.18, and 38.29 are associated with nickel oxalate phase^31^. Several other peaks are also present in the pattern, which, as shown in Fig. 8, are attributed to compounds containing other metals. At pH 2-3, it is possible for cobalt metal and even manganese and lithium to react with oxalic acid. According to other studies^31–33^, the formation of a small amount of cobalt oxalate and other metals has caused the appearance of a number of peaks in the XRD pattern. Table 4 shows the amount of important elements in the precipitation obtained from this step.

**Table 3:** Metal concentrations (%) in the solution obtained from each step.

Metal	Nickel separation		Cobalt and Manganese precipitation	Ammonia addition	Lithium separation
	pH = 2	pH = 3			
Li	84	86	81	78	5
Co	73	70	9	10	8
Mn	77	76	8	11	7
Ni	10	16	9	7	7

**Table 4:** Amount of important elements present in the sediment obtained from different steps.

Element	Nickel oxalate precipitation	Cobalt and manganese hydroxide precipitation	Lithium carbonate precipitation
Ni	28.2	4.1	1.3
Co	3.7	19.2	2.1
Mn	1.9	27.4	2.9
Li	0.6	0.7	16.2
C	18.6	1.8	17.5
O	44.1	39.7	58.2

Figure 8 shows the SEM image, EDX, and MAP analysis of the precipitated particles. The amount of cobalt and manganese is low, so, due to the high nickel content, this precipitate contains nickel compounds. As demonstrated in Fig. 7, the NiC_2_O_4_ (nickel oxalate) is the dominant compound.

Two methods were investigated for the precipitation of manganese and cobalt. The first method was employed to remove Mn and Co metals from the remaining sodium hydroxide solution. Therefore, changing the pH and increasing it to 8 with the help of sodium hydroxide at this stage plays a significant role in the formation of the precipitate^34^. By adding sodium hydroxide to the solution containing manganese, a white solid precipitates. This precipitate contains pyrochroite Mn(OH)_2_^35^:

10 $${\text{Mn}}^{{2 + }} {\text{ + 2OH}}^{ - } {\text{ = Mn(OH)}}_{{2}}$$

On the other hand, their general reactions can be expressed as follows:

11 $$\mathrm{NH}_{2}\mathrm{CH}_{2} \mathrm{COOH} + \mathrm{NaOH} \Rightarrow \mathrm{NH}_{2} \mathrm{CH}_{2} \mathrm{COONa} + \mathrm{H}_{2} \mathrm{O}$$

12 $${\text{Mn}}^{2 + } + 2{\text{NaOH}} + 2{\text{H}}_{2} {\text{O}} \Rightarrow {\text{Na}}_{2} \left[ {{\text{Mn}}\left( {{\text{OH}}} \right)_{4} } \right] + 2{\text{H}}^{ + }$$

13 $${\text{Co}}^{2 + } + 2{\text{NaOH}} \Rightarrow {\text{Co}}\left( {{\text{OH}}} \right)_{2} + 2{\text{Na}}^{ + }$$

According to Ou et al.^36^, the chemical precipitation method is suitable for recycling spent lithium-ion batteries and uses precipitating agents to precipitate the metals. Contestabile et al.^37^, in this process, recovered the cobalt dissolved in the solution in the form of cobalt hydroxide, and the pH changes play a significant role in the formation of the precipitate and recovery.

In the Pourbaix diagram of manganese and cobalt compounds in water at ambient temperature and in the presence of the OH- ligand, at pH levels less than 7.5, manganese is in the soluble form and can precipitate from the acidic solution by adjusting the pH (alkalization) or altering the oxidation conditions. Therefore, if the pH is increased, manganese will precipitate as hydroxide. Practically all manganese should be precipitated at a pH higher than about 8. However, under the influence of a strong oxidizing agent (such as hydrogen peroxide), manganese may precipitate as MnO_2_ regardless of the final pH value^38–40^. In this study, since no strong oxidizing agent was used, there is no possibility of manganese oxides forming.

Similar to manganese, cobalt precipitation in the form of hydroxide usually occurs at pH values ​​around 8.5. Increasing the Eh of the solution can also precipitate this metal at lower pH values ​​(less than 6.5). Pourbaix diagrams show cobalt precipitation as Co(OH)_2_ and Co(OH)_3_ with increasing pH or Eh in the presence of OH^−^^40,41^.

**Fig. 9:**
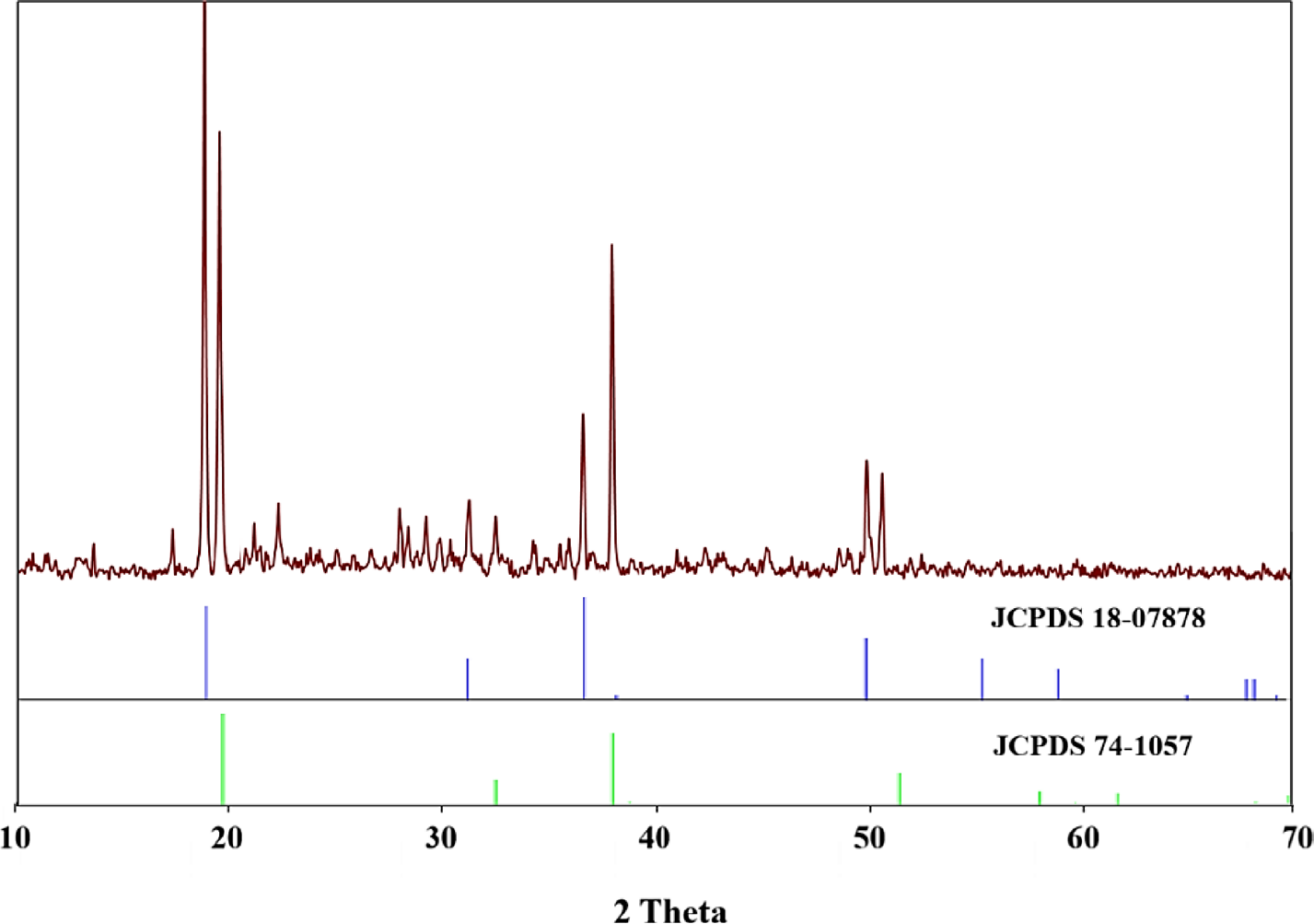
XRD pattern of the precipitate resulting from the addition of sodium hydroxide (Mn(OH)_2_: JCPDS 18-0787, and Co(OH)_2_: JCPDS 74-1057).

**Fig. 10:**
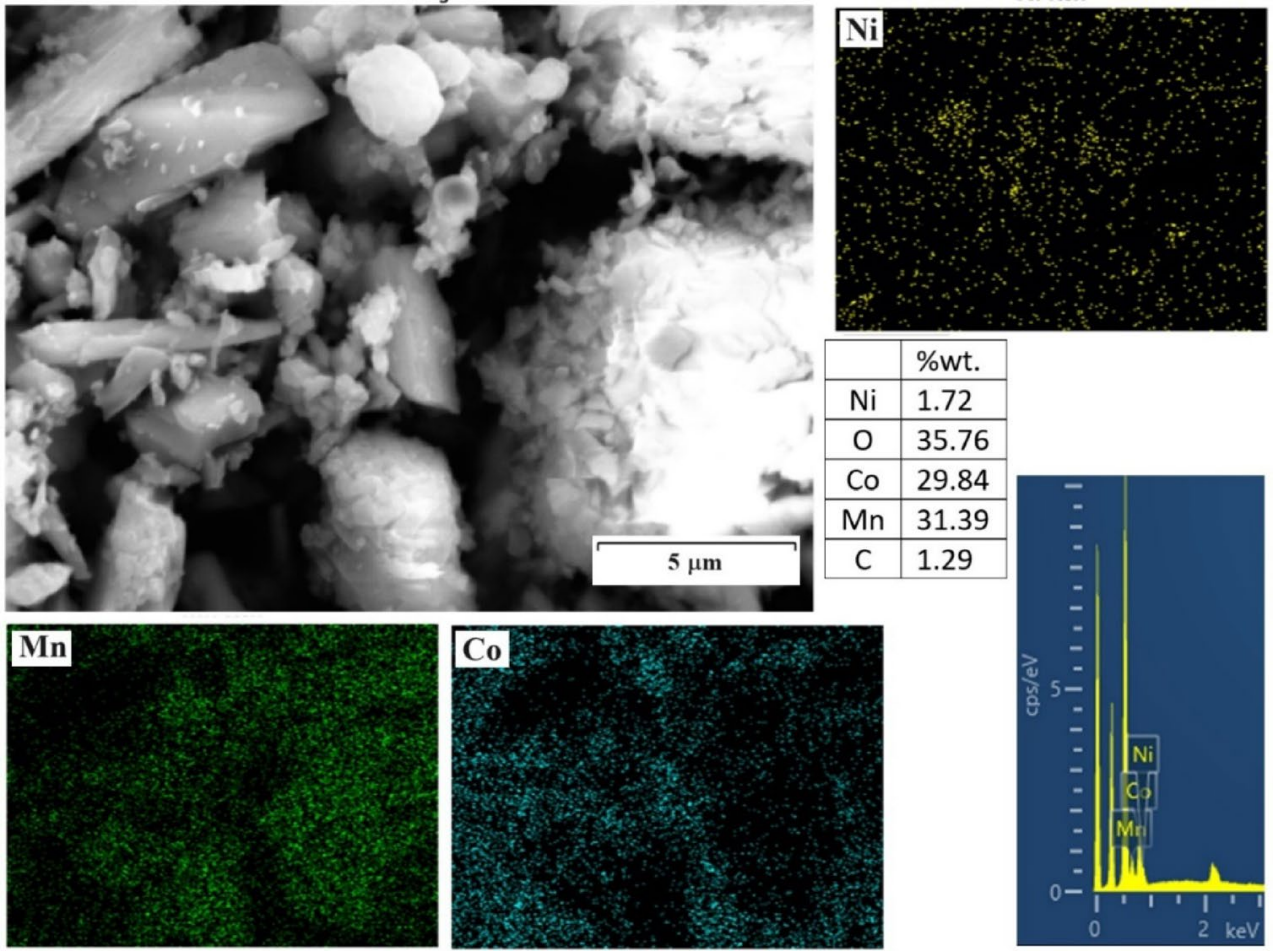
SEM image, MAP analysis, and EDX analysis of the particles precipitated by soda.

According to Table 3, a significant amount of cobalt and manganese in the solution is reduced and precipitated. Figure 9 shows the XRD spectra of cobalt and manganese precipitation. The peaks at angles 20.21, 36.82, 39.04, and 50.62 are related to the cobalt hydroxide phase planes according to JCPDS 74-1057. Additionally, the peaks identified at angles of 18.71, 31.11, 36.57, and 49.85 are attributed to manganese hydroxide, as per JCPDS 18-0787. Other peaks are related to the presence of other metals, which are identified in Fig. 10. Based on previous studies, nickel can precipitate as nickel hydroxide if present at pH 8. Of course, some lithium hydroxide will probably also precipitate^42–44^. Table 4 shows the amount of important elements present in the precipitation resulting from this step.

SEM was used to determine the morphology of the precipitate particles. Figure 10 shows the results of EDX and map analysis in addition to the SEM image. The distribution of metals proves that cobalt and manganese are more abundant than nickel. Given that a large amount of nickel was separated in the previous step, a small amount of it was detected in this step. Since the hydrogen cannot be detected, the large amount of oxygen refers to the formation of cobalt hydroxide (Co(OH)_2_) and manganese hydroxide (Mn(OH)_2_). In the second method of precipitation of manganese and cobalt, by adding ammonia to the solution and increasing the pH of the solution to 9, the precipitation of manganese and cobalt began. The result of the analysis of the solution after separation of the precipitate is shown in Table 3. As can be seen, a small amount of cobalt, manganese, and nickel remains in the solution. It is expected that a large amount of these metals will be present in the precipitate.

The following possible reactions can occur during precipitation^30^.

14 $$\mathrm{NH}_{2} \mathrm{CH}_{2} \mathrm{COOH} + \mathrm{NH}_{3} \Rightarrow \mathrm{NH}_{2} \mathrm{CH}_{2} \mathrm{COONH}_{4}$$

15 $${\text{Mn}} + {\text{NH}}_{3} + {\text{H}}_{2} {\text{O}} \Rightarrow {\text{Mn}}\left( {{\text{OH}}} \right) + {\text{NH}}_{4}$$

The manganese precipitate is insoluble in excess ammonia, but it is soluble in solutions containing ammonium salts. The precipitate undergoes facile oxidation by atmospheric oxygen, yielding manganese(III) or manganese(IV) species that impart a characteristic brown coloration to the solid.

In the case of cobalt precipitation with ammonia, the excess concentrated ammonia reacts with the cobalt(II) ion to form a metal complex ion, the hexaamine cobalt(II) ion:

16 $${\text{Co}}_{(aq)}^{2 + } + 6{\text{NH}}_{3(aq)} \Rightarrow \left[ {{\text{Co}}\left( {{\text{NH}}_{3} } \right)_{6} } \right]^{2 + }$$

If there is not enough ammonia, the reaction leads to the precipitation of a salt, Co(OH)NO_3_ or Co(OH)_2_, which can precipitate with other metal ions to form hydroxide precipitates.

17 $${\text{Co}}^{2 + } + {\text{OH}}_{(aq)}^{ - } + {\text{NO}}_{3(aq)}^{ - } \Rightarrow {\text{Co}}\left( {{\text{OH}}} \right){\text{NO}}_{3(s)}$$

According to the Pourbaix diagram of manganese and cobalt compounds in water and ambient temperature, in the presence of NH^4+^ ligand, similar to the presence of OH^-^ ligand, cobalt and manganese precipitate at alkaline pH. Cobalt precipitation occurs as Co(OH)_2_ and Co(OH)_3_ with an increase in pH to more than 8.5 or high Eh in the presence of NH^4+^ ligand^40^.

According to the comparison of the results of Table 3 and the volatility of ammonia during the experiment and the economic nature of the first method, the first method is the most suitable, and a larger amount of precipitate is extracted here.

For lithium separation, given that the percentage of lithium dissolution in glycine acid was very high, this number indicates that the lithium has a high solubility in glycine solution. Adding sodium carbonate increased the pH of the solution to 9. Table 3 shows the results of the ICP analysis of the solution. As can be seen, the amount of metals in the remaining solution has decreased significantly. Of course, the largest change is related to the reduction of lithium compared to Table 3 (precipitation of manganese and cobalt).

The following reactions can be considered for the precipitation of lithium by sodium carbonate:

18 $$2{\text{NH}}_{2} {\text{CH}}_{2} {\text{COOH}} + {\text{Na}}_{2} {\text{CO}}_{3} \Rightarrow 2{\text{NH}}_{2} {\text{CH}}_{2} {\text{COONa}} + {\text{CO}}_{2} + {\text{H}}_{2} {\text{O}}$$

19 $$2{\text{Li}} + {\text{Na}}_{2} {\text{CO}}_{3} \Rightarrow {\text{Li}}_{2} {\text{CO}}_{3} + 2{\text{Na}}$$

Chen et al.^45^ presented a method that is both effective and economical for recovering lithium from lithium-ion batteries, achieving a recovery efficiency of 91.1% for lithium under optimized experimental conditions. The lithium leaching efficiency can be increased to 94.6% with an acidic solution. However, the recovery of lithium from used batteries has rarely been reported. Zhao et al.^46^ studied the recovery of lithium carbonate from lithium chloride solution using sodium carbonate and ultrasound. Lithium sulfate solutions from the cathode waste of lithium-ion batteries were prepared at 70 °C.

**Fig. 11:**
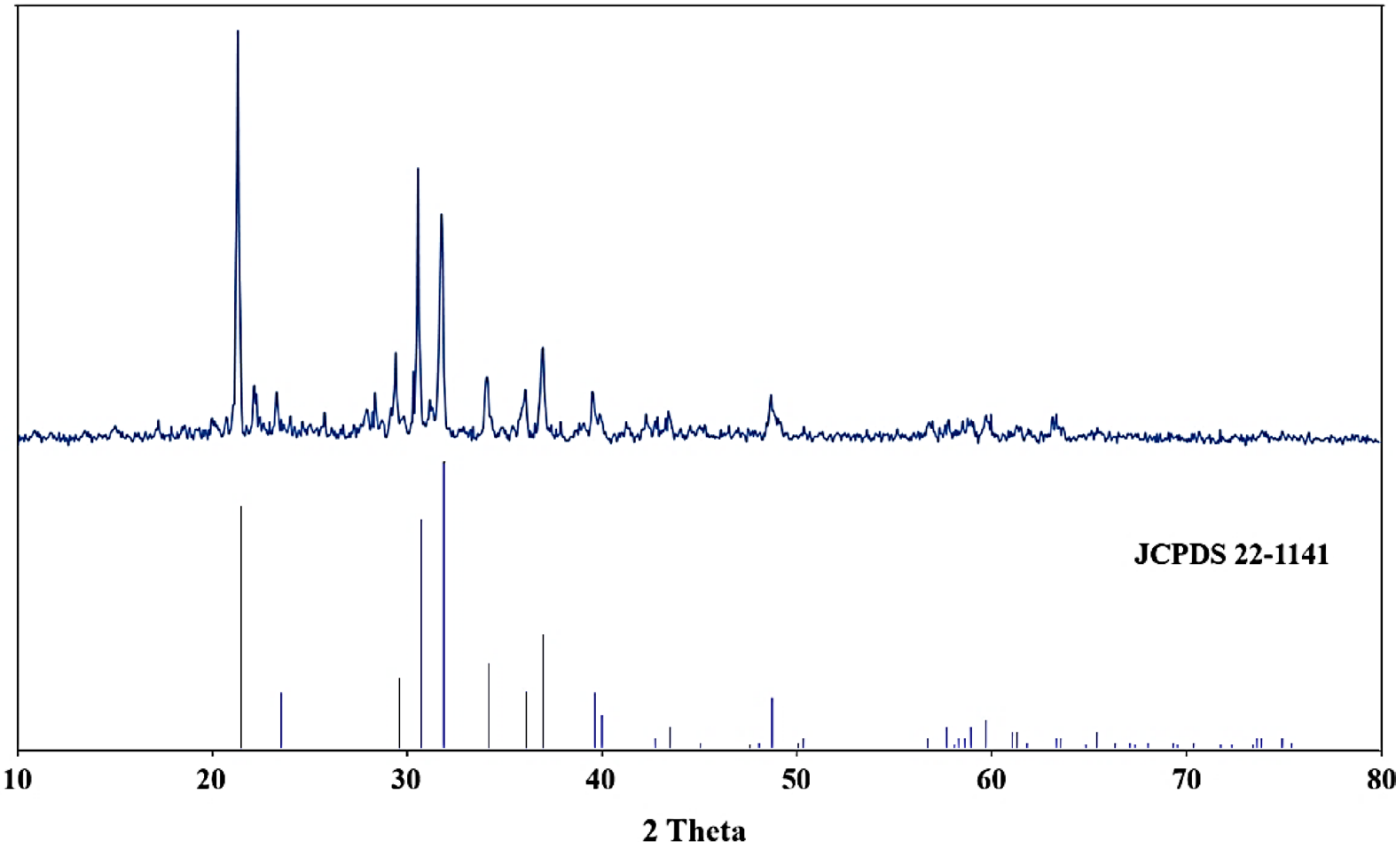
XRD pattern of the precipitate of Li_2_CO_3_ obtained by adding sodium carbonate to the solution (JCPDS 22-1141).

Jiang et al.^47^ investigated the production of Li_2_CO_3_ from lithium salt solution by a laboratory-scale electrodialysis system. The salt solution was first contacted with Na_2_CO_3_ to reduce Ca^+2^ and Mg^+2^. After that, a conventional electrodialysis process was performed to increase the Li^+^ concentration. After the Li_2_CO_3_ precipitation, a secondary crystallization step was performed to increase the purity of the powder. Figure 11 shows the XRD pattern of the precipitate from the lithium separation step. The peaks observed at angles of 21.19, 23.32, 29.27, 30.57, and 31.66° are related to the lithium carbonate phase planes according to JCPDS 22–1141. Due to the removal of most metals, there is very little impurity in this precipitate. However, small peaks that could be related to nickel and cobalt carbonate can be detected^48,49^. The amount of important elements in the precipitation obtained from this step can be seen in Table 4.

**Fig. 12:**
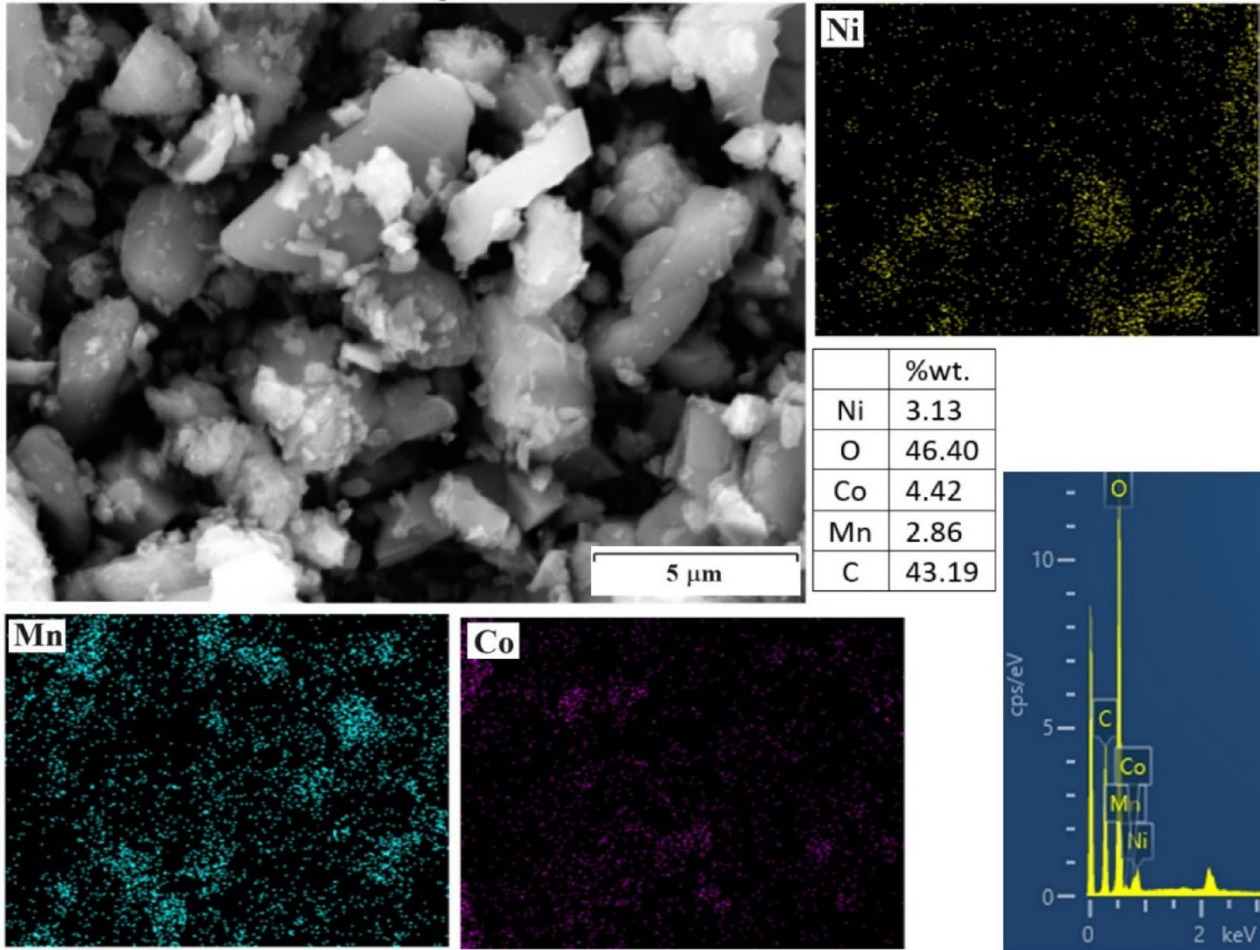
SEM image, MAP analysis, and EDX analysis of deposited particles.

Figure 12 shows the SEM image, the presence, and distribution of various metals in the precipitate of the lithium separation stage. The results prove the presence of small amounts of metals manganese, cobalt, and nickel in the concentration of oxygen and carbon. Considering the inability of EDX to detect lithium, which has been observed in similar studies^43^, and as determined by the XRD pattern, lithium carbonate is present in the precipitate. Finally, the calculations showed that the recovery rates of lithium, cobalt, manganese, and nickel were 96.7, 93.5, 94.2, and 92.8%, respectively.

## Conclusion

In this project, dissolution and separation of nickel, manganese, cobalt, and lithium in the battery were carried out. The results are as follows:

1. The valuable metals nickel, manganese, cobalt, and lithium in the lithium-ion battery are well dissolved in glycine acid with a concentration of 1 molar and pH = 4. Of course, the effect of important parameters such as glycine concentration, time, and temperature cannot be ignored.
2. By reducing the pH of the solution with oxalic acid to 2, at 60 °C for 60 min, nickel can be precipitated as nickel oxalate (NiC_2_O_4_). In this case, manganese, cobalt, and lithium will remain in solution and will not precipitate.
3. Cobalt and manganese can be precipitated by adding an alkaline substance such as soda or ammonia and increasing the pH to about 8. In this case, manganese hydroxide and cobalt hydroxide can be precipitated, especially when 5 M sodium hydroxide is added at pH 8 and 25 °C.
4. After the separation of nickel, manganese, and cobalt, the solution will contain a large amount of lithium. The precipitation of lithium carbonate (Li_2_CO_3_) is carried out by adding sodium carbonate at pH 9 and 95 °C.

## Data Availability

The datasets generated during and/or analyzed during the current study are available from the corresponding author upon reasonable request.
